# The Influence of Communication on College Students’ Self–Other Risk Perceptions of COVID-19: A Comparative Study of China and the United States

**DOI:** 10.3390/ijerph182312491

**Published:** 2021-11-27

**Authors:** Yi Yang, Ru-De Liu, Yi Ding, Jia Wang, Wei Hong, Ying Wu

**Affiliations:** 1 Beijing Key Laboratory of Applied Experimental Psychology, National Demonstration Center for Experimental Psychology Education, Faculty of Psychology, Beijing Normal University, Beijing 100875, China; yi.yang@mail.bnu.edu.cn (Y.Y.); psyhongwei@163.com (W.H.); 2 Graduate School of Education, Fordham University, New York, NY 10023, USA; yding4@fordham.edu (Y.D.); ywu135@fordham.edu (Y.W.); 3 Teachers’ College, Beijing Union University, Beijing 100874, China; wangjia@mail.bnu.edu.cn

**Keywords:** interpersonal communication, media exposure, risk perception, comparative study, college students, COVID-19

## Abstract

This study aimed to explore cross-country differences in the characteristics and determinations of self–other risk perceptions of the COVID-19 pandemic. We distinguished perceived risk to self from perceived risk to others and subdivided risk perceptions into three levels: personal, group, and societal. We focused on the differential impact of multiple communication channels (i.e., interpersonal communication, traditional media exposure, and new media exposure) on risk perceptions at the three levels. A sample of 790 college students completed self-report online questionnaires from May to June 2020, including 498 in China and 292 in the United States. The results showed an “ascending pattern,” revealing that participants perceived higher levels of risk to others than to themselves. In addition, U.S. college students perceived higher risks of COVID-19 than Chinese college students at all levels. As for the relations between communication and risk perceptions, the results revealed that interpersonal communication and traditional media exposure were more effective with Chinese participants, whereas new media exposure was more effective with U.S. participants. Specifically, interpersonal communication was positively associated with risk perceptions at three levels, and the magnitude of the effect was higher in the Chinese group than in the U.S. group. Traditional media exposure increased societal risk perception only for Chinese college students, and new media exposure increased societal risk perception only for U.S. college students. Our findings provide theoretical implications for the characteristics and forming mechanisms of risk perceptions and also provide practical implications for policymakers in the two countries to implement effective measures to foster individuals’ risk perceptions in relation to preventive behaviors.

## 1. Introduction

The novel coronavirus disease 2019 (COVID-19) has spread rapidly around the world and was assessed as a global pandemic on 11 March 2020 by the World Health Organization [1]. Due to the high morbidity, high mortality, and widespread transmission modes of COVID-19, the general population has faced uncertainty and instability for a prolonged period and has reported increased levels of stress, anxiety, and depression related to the pandemic [2,3]. Compared to other health threats, such as influenza, H1N1, cancer, heart attack, and food poisoning, participants have reported higher levels of worry and perceived risk associated with COVID-19 [4,5,6].

Individuals’ perceived infection risks have essential positive effects on their adherence to precaution guidelines and preventive behaviors [7,8], such as keeping social distance, wearing masks, and washing hands. COVID-19 is an infectious disease that has various transmission modes including contact, droplet, and airborne transmission. Thus, not only the perception of personal infection risk but also the perceived risk to others might contribute to preventive behaviors. If someone perceives that their neighbors are at high risk of infection, they are likely to maintain social distance from those neighbors. Previous studies have distinguished perceived risk to oneself from perceived risk to others in other health issues, such as H1N1 flu [9] and skin cancer [10], and found that people perceive higher levels of risk to others than to themselves. Exploring self–other differences in risk perceptions of COVID-19 was one of the main purposes of this study.

Individuals tend to seek information and communication when facing unknown diseases. Multiple information sources play an important role in individuals’ risk perceptions [11]. Distinct information channels can be classified by channel types, such as traditional media, new media, and interpersonal communication, which may have different effects on risk perceptions to oneself and to others [9,10]. Identifying the determinants of self–other risk perceptions of COVID-19 might be helpful to provide psychological and social perspectives for policymakers and service providers to implement effective preventive measures.

China and the United States are two of the countries most affected by the COVID-19 pandemic, and they have fundamental cultural differences. The former is a typical collectivist country, and the latter is a typical individualistic country. According to existing literature, there might also be cultural differences in risk perceptions [12,13,14]. The present study took a comparative perspective to explore the characteristics and determinants of risk perceptions to oneself and to others between China and the United States.

### 1.1. Self–Other Risk Perceptions of COVID-19

Risk perceptions, also known as risk judgments, comprise two dimensions. The cognitive dimension refers to the individual’s subjective assessment of the likelihood of infection, and the affective dimension refers to concern or worry about the risk [15]. When evaluating the risk of a health crisis, people may differ in estimating the risk to themselves and the risk to others and show bias about their vulnerability to harm. The “optimism bias” can be defined as individuals’ unrealistic beliefs that they are less likely than others to experience health problems [16,17]. Empirical research has provided supporting evidence for this phenomenon, showing that individuals perceived higher levels of risk to others than to themselves for many health issues, such as heart disease, skin cancer, smoking, AIDS, SARS, H1N1, water contaminants, radon, and COVID-19 [9,10,13,15,18,19,20].

Moreover, the “optimistic bias” becomes greater when the psychological, social, or physical distance increases between oneself and the comparison group [21]. For example, Wu and Lin [22] conducted an experimental study and found that the differences between perceived risk to self and to others increased when people compared themselves with a more distant individual. Han et al. [9] separately measured individuals’ perceived risk to themselves, their peers, average people in their own countries, and average people living abroad and found an “ascending pattern” in which their perceived risk increased when the social distance between self and others increased.

However, most studies lack a clear conceptual and operational definition of “risk perception of others.” When comparing self and other risk perceptions, different researchers have different focuses and measures. Previous studies compared individuals’ perceived risk to themselves with numerous targets, such as their relatives [20], classmates [16], average people of their age and sex [23,24], average people from their own country [19,25], and average people from other countries [9]. These studies lacked a clear subdivision of these numerous target individuals who varied in closeness. Some studies combined the targets and simultaneously measured different targets together [20]. Some studies did not assess perceived risk to self and others separately [26]. One study about skin cancer clarified the definition of risk perceptions to self and to others and subdivided the risk perception into three levels: personal, group, and societal [10]. It was reported that the perceived risk increased when the reference’s distance from the self got further away.

There is a lack of empirical study to examine the self–other difference in risk perception of COVID-19, especially with more specific subcategories of perceived risk to others. The present study subdivided risk perceptions into three levels, including the personal level (self), group level (peers), and societal level (average people in the country), and aimed to explore the characteristics of self–other risk perceptions of COVID-19 among college students. We hypothesized that:

**Hypothesis** **H1:** *Individuals perceive a higher risk of COVID-19 for others than for themselves. The perceived risk for others might ascend when the reference’s distance (i.e., group, societal) becomes further away from the personal level.*

### 1.2. Cross-Country Differences in Self–Other Risk Perceptions

Nationality, as a type of demographic variable, has been found to be associated with risk perceptions according to previous studies [12,14,27,28]. Different nations have different mainstream cultural values. China and the United States, as two of the countries most affected by COVID-19, were chosen for their cultural differences. The former is known as a typical collectivist culture, and the latter is known as a typical individualistic culture. Previous studies have shown that collectivism can serve as a buffer against psychological threats and maladjustment related to infectious disease [29,30]. People with a higher collectivist orientation have a stronger sense of social responsibility and tend to show greater cooperation, conscientiousness, and conformity. However, those with a higher individualist orientation are more likely to emphasize personal independence and freedom [31]. Thus, compared with individualists, collectivists are more likely to comply with government mandates, such as staying at home, to avoid potential harm to themselves and others. Moreover, social support and emotional connection valued by a collectivistic culture provide a defense against high risk perception and psychological maladjustment [30]. Based on the above evidence, Americans may perceive higher risks of COVID-19 than Chinese people.

On the other hand, objectively speaking, the number of total cases and deaths due to COVID-19 reported in the U.S. has been much higher than the number reported in China. Official data from the World Health Organization (WHO) showed 84,588 confirmed cases in China, including 4645 deaths, and 1,764,252 confirmed cases in the United States, including 105,561 deaths, between 31 December 2019, and 1 June 2020. Compared with China, the COVID-19 situation has been more severe in the United States. Thus, the pandemic may have created more concerns and worries for Americans. We hypothesized that:

**Hypothesis** **H2:** *Americans perceive a higher risk of COVID-19 at each of the three levels (i.e., personal, group, societal) than Chinese.*

### 1.3. The Influence of Communication on Self–Other Risk Perceptions

When facing unknown diseases, people may rely on multiple communication sources to obtain related information and evaluate the level of risk to themselves and others [11]. Distinct communication channels can be classified by channel types, including interpersonal communication, traditional media, and new media. Each information source has distinctive characteristics that may differentially affect the perceived risk to self and others [32]. The present study aimed to identify the relation between multiple information sources and risk perceptions at different reference levels (i.e., personal, group, and societal).

#### 1.3.1. Interpersonal Communication

Interpersonal communication is an essential channel for individuals to obtain information about health issues. According to the social amplification of risk framework [33], interpersonal interactions can intensify public concerns and responses to risk events. The existing literature provides mixed evidence for the influence of interpersonal communication on perceived risk to self and others. On the one hand, it is argued that interpersonal communication leads to increased risk perception only to oneself rather than to others [34]. It is plausible that when people talk about risk-related information with others (e.g., family, friends, neighbors), such conversational interaction is highly personally relevant and forces people to consider risks to themselves. Previous studies have found that interpersonal communication positively predicted the personal risk perception of tuberculosis [35], breast cancer [36], the Fukushima nuclear accident [11], and haze [37]. On the other hand, it is suggested that interpersonal communication can also increase the perceived risk to others. It is possible that talking about health risks with others makes people realize that individual risks can be collectively shared [38].

Some studies integrated the above views and found that interpersonal communication positively correlated with personal and societal risk perception of skin cancer [10]. Han et al. [9] conducted a comparative study in the United States and China and found that interpersonal discussions about H1N1 were positively associated with risk perceptions at the personal, group, and societal levels in both countries. To our knowledge, none of the existing studies examined the influence of interpersonal communication on self–other risk perceptions of COVID-19. Based on the above evidence for the positive association between interpersonal communication and self–other risk perception of other health threats, we hypothesized that:

**Hypothesis** **H3a:** *Interpersonal communication is positively associated with risk perception at all reference levels (i.e., personal, group, societal) in both China and the United States.*

#### 1.3.2. Mass Media Exposure

In addition to interpersonal communication, mass media, accompanied by vivid images or videos, provide a convenient public channel for people to obtain more detailed information about health issues. Media tend to highlight the risks and negative consequences of health issues to the public [39,40], thus increasing individuals’ risk perceptions. Tyler and Cook [32] distinguished the media effect on risk perceptions at personal and societal levels and proposed the impersonal impact hypothesis. The impersonal impact hypothesis suggests that mass media exposure heightens risk perception only at the more general collective level rather than at the personal level [32]. Considerable empirical studies have provided supporting evidence for this hypothesis and shown that personal risk perception is mainly affected by personal experience and interpersonal communication that is highly personally relevant rather than by media exposure. Media exposure mainly influences individuals’ perceived risks to society [10,19,37,41].

The impersonal effects of media exposure may be due to the fact that mass media content is more relevant to the whole societal situation, such as the total confirmed cases and deaths in a country. Such general statistical information helps people form general judgments about the base rate of infection but may not increase people’s perception of their own risks [34]. An empirical study showed that knowledge of the general crime rate did not correlate with individuals’ fear of personal crime victimization [42]. Perceived personal risks may be the result of personal related and purposeful information-gathering activities, such as interpersonal communication [15]. The above evidence helps to explain why media exposure contributes only to increased societal risk perception.

When exploring the media effects, earlier studies have focused on traditional media, such as television, newspapers, and magazines [10,19]. With the rapid development of the internet, researchers have begun to pay attention to the effects of new media, such as portal websites and social media [11,43]. In traditional media channels, individuals mainly play the role of passive information receivers, whereas in new media channels, individuals act as both information receivers and providers. Such two-way processes may improve the accessibility of risk-related information, thus increasing risk perceptions [44]. However, social media are more likely to provide unofficial or even false and exaggerated information [45,46]. The health-related information on traditional media is relatively official and authoritative. Considering the differences in the characteristics of traditional media and new media, it is worthwhile to distinguish them and separately examine the effects of traditional and new media exposure on self–other risk perceptions. Based on the above discussion, we hypothesized that:

**Hypothesis** **H3b:** *Traditional and new media exposure are positively associated with risk perception at the societal level but not at the personal or group level in both China and the United States.*

### 1.4. Cross-Country Differences in the Influence of Communication on Self–Other Risk Perceptions

Most previous studies have explored the effects of multiple information sources on risk perceptions in a specific country. The results are difficult to generalize to countries with different cultures. The media channels may be similar in different countries, but the importance and magnitude of each media effect may have cross-country differences. Han et al. [9] examined the effects of communication factors on risk perceptions of H1N1 in China and the United States and found the strongest predictors in each country by comparing country-specific beta weights in the regression models. However, the results did not further compare the magnitude of media effect across countries. The present study constructed interaction terms between multiple communication factors and nationality to compare the magnitude of media effect between China and the United States. This can help us to better understand the formation and influencing factors of risk perceptions from a global perspective and provide scientific guidance for policymakers in different countries to implement effective preventive measures.

#### 1.4.1. Interpersonal Communication

Compared with individualists, collectivists tend to emphasize maintaining close social relationships and be more willing to accept the opinions and views of others [47]. Chinese people who are influenced mostly by collectivism may rely more on interpersonal communication compared with Americans who are influenced mostly by individualism. In other words, Chinese people may pay more attention to the sharing or exchange of information from those around them, which effectively increases perceived risk to self and collective others. Therefore, with regard to the cross-country differences in the influence of interpersonal communication on self–other risk perceptions, we hypothesized that:

**Hypothesis** **H4a:** *Compared with the United States, interpersonal communication has a more effective positive impact on risk perceptions at three levels in China.*

#### 1.4.2. Mass Media Exposure

Both traditional and new media exposure may increase societal risk perception in China and the United States, but the magnitude of each media effect may vary across countries. On the one hand, in China, traditional media, such as CCTV news, which is considered the most official and authoritative television news, report COVID-19-related news every day and thus gain more public trust and attention than other media sources [6]. However, in the United States, there is substantial political polarization about COVID-19-related information [48]. According to previous studies [48,49], given a choice between CNN/MSNBC or Fox News, Democrats tended to prefer CNN or MSNBC, whereas Republicans reported being more likely to watch Fox News, and Third-Party/Independents were equally divided but tended not to watch those cable news sources. Different traditional media channels have distinct attitudes towards risk perceptions and protective behaviors related to the COVID-19 pandemic [50]. Such mixed preferences among the American public may attenuate the influence of traditional media. Thus, we hypothesized that:

**Hypothesis** **H4b:** *Compared with the United States, traditional media exposure has a more effective positive impact on societal risk perception in China.*

On the other hand, Zhong [6] investigated multiple information sources and found that Chinese people had the lowest trust and confidence in information disseminated on the internet and in social media (e.g., WeChat and Weibo). In the early stages of the pandemic, rumors and misinformation were spreading on new media, especially about the origins of the SARS-CoV-2 virus, which reduced the credibility of new media. Later, China implemented internet control of COVID-19-related information to filter out improper public statements and comments, which may have weakened the increasing effect of new media on risk perception. However, in the United States, there was relatively free access for the public to express opinions and make comments via new media, regardless of whether information had been validated. Cinelli et al. [51] identified the amplification of rumors and inaccurate information on social media platforms, including YouTube, Twitter, Instagram, Reddit, and Gab, which may have led to increased risk perception in the United States. Thus, we hypothesized that:

**Hypothesis** **H4c:** *Compared with China, new media exposure has a more effective positive impact on societal risk perception in the United States.*

### 1.5. The Present Study

**Figure 1:**
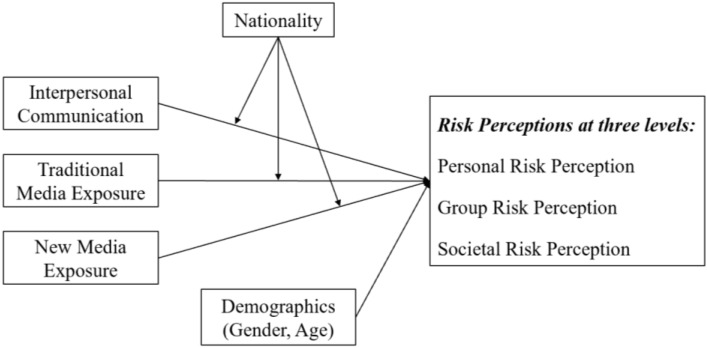
Conceptual model of national differences in the influence of communication factors on risk perceptions.

There were three primary aims of this study: (a) to investigate the characteristics of self–other risk perceptions of COVID-19 at three levels (i.e., personal, group, and societal levels); (b) to examine the effects of different information sources on self–other risk perceptions, and (c) to explore the cross-country differences in the characteristics and determinants of self–other risk perceptions between college students in China and the United States. The conceptual model is shown in Figure 1.

By taking a global and comparative perspective, the present study can help us better understand the characteristics, formation, and cultural differences of self–other risk perceptions of COVID-19. It can also provide scientific guidance for policymakers in different countries to implement effective measures for shaping expected social responses.

## 2. Methods

### 2.1. Participants

In this study, data were collected via online survey platforms (i.e., Wenjuanxing in China and Qualtrics in the United States) from May to June 2020. A total of 790 participants completed self-report online questionnaires, including 498 in China and 292 in the United States. Participants were college students enrolled at Chinese/American universities and residing in China/the United States during the initial peak of COVID-19. Among all participants, 553 (70.0%) were female, 231 (29.2%) were male, and 6 (0.8%) reported a non-binary gender. The average age of the students was 20.83 years (*SD* = 2.89), with a range from 17 to 30 years.

### 2.2. Procedures

The present study was approved by the Research Ethics Committee of the Faculty of the researchers’ institutes in China and the United States. Consent was obtained from all participating students. Participants volunteered to complete an online survey that included measures of self–other risk perceptions of COVID-19, the frequency of COVID-19-related information communication during the initial peak of the pandemic, and demographic characteristics. It took about 10 min to complete the online survey. Then, participants received compensation such as a special postcard or a gift card through raffles.

### 2.3. Measures

#### 2.3.1. Self–Other Risk Perceptions of COVID-19

Risk perceptions of COVID-19 were measured by 12 items adapted from previous studies [9,10,32]. This factor was assessed at three different levels (personal, group, and societal). Each level contained four items. For perceptions of personal risk, participants rated the items on a 5-point Likert scale, indicating “How important is COVID-19 to you?” (1 = not at all, 5 = very important); “How much risk do you perceive from COVID-19?” (1 = none at all, 5 = a great deal); “How likely is it that you would be infected with COVID-19?” (1 = very unlikely, 5 = very likely); “How worried are you about yourself being infected with COVID-19 in the future?” (1 = not at all, 5 = very worried). The same four items were reworded to measure perceptions of risk to their peers (group risk) or to the average person in their own countries (societal risk). Higher scores indicated higher levels of risk perceptions during the initial peak of the COVID-19 pandemic. In the present study, this scale showed adequate reliability for all items (Cronbach’s α = 0.87) as well as at different reference levels (personal risk, α = 0.78; group risk, α = 0.76; societal risk, α = 0.73).

#### 2.3.2. Interpersonal Communication

Interpersonal communication was measured by a 5-point Likert scale containing five items [9,52]. Participants responded to “How much have you discussed about COVID-19 with the following others during the peak time of COVID-19?” (1 = not at all, 5 = a great deal). Target others were (a) peers (e.g., classmates, friends), (b) family, (c) professors, (d) health care professionals (e.g., doctors, nurses), and (e) other people. Higher scores indicated higher levels of interpersonal communication about COVID-19 during the initial peak of the COVID-19 pandemic. This scale showed good reliability in the present study (Cronbach’s α = 0.76).

#### 2.3.3. Mass Media Exposure

Mass media exposure was measured by a 5-point Likert scale containing five items. This factor was assessed via two types of media exposure. The first two items measured traditional media exposure [10,52] and the last three items measured new media exposure [9]. Participants responded to “How frequently have you seen, read, or heard anything about COVID-19 from the following media sources during the peak time of COVID-19” (1 = never, 5 = always). Target media sources were (a) television news, documentaries, and current affairs; (b) television entertainment programs (e.g., soap operas, sitcoms, drama, movies); (c) portal websites, news websites, or online versions of traditional media; (d) social media (e.g., Facebook); (e) official university websites or health care/medical websites. Higher scores indicated higher levels of media exposure to COVID-19-related information during the initial peak of the COVID-19 pandemic. This scale showed good reliability in this study (Cronbach’s α = 0.72).

### 2.4. Data Analysis

Descriptive analyses and Pearson correlations were calculated using SPSS 25.0. To test the “ascending pattern” of risk perceptions, repeated measures ANOVA and within-subjects contrasts tests were conducted to compare risk perceptions across three levels among college students in each country. To examine country differences in risk perception at each level, independent *t*-tests were conducted to compare risk perceptions between college students in China and the United States.

Three separate hierarchical multiple regression analyses were used to examine the effects of COVID-19-related communication predictors on risk perceptions at three levels and the moderating role of nationality. When generating interactions terms, communications variables were calculated using standardized scores, and nationality was defined as a dummy variable (0 = China, 1 = USA). Significant interactions were further explored and graphed using simple slope analyses.

## 3. Results

### 3.1. Descriptive Statistics and Correlations

Table 1 shows the means, standard deviations, and correlations between study variables. Gender and age were correlated with other study variables, which indicated that gender and age needed to be considered as control variables in subsequent analyses. For the main variables, nationality was correlated with other variables, which suggested country differences. Interpersonal communication, traditional media exposure, and new media exposure were positively correlated with each other. Risk perceptions at three levels were positively correlated with each other. Interpersonal communication and new media exposure were positively correlated with risk perceptions at all three levels. Traditional media exposure was positively correlated with risk perception only at the societal level.

### 3.2. Self–Other Risk Perceptions of COVID-19

**Figure 2:**
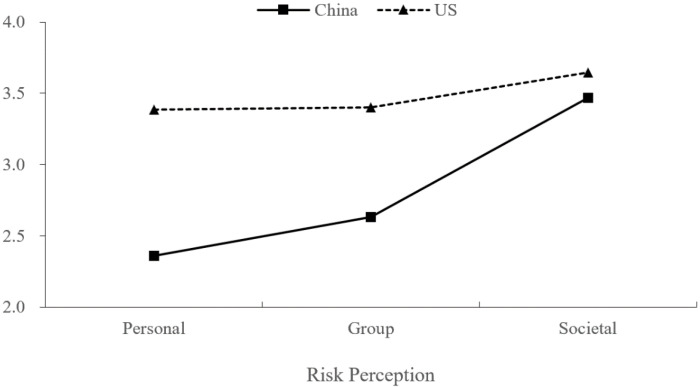
The “ascending pattern” of risk perceptions along the personal-group-societal levels in two countries.

As shown in Table 2, for Chinese participants, the perception of societal risk was significantly higher than that of group risk, which was further higher than that of personal risk. For U.S. participants, the perception of societal risk was significantly higher than that of group risk. The perception of group risk tended to be higher than that of personal risk, although it was not statistically significant. Figure 2 presents the “ascending pattern” of risk perceptions along the personal–group–societal levels among participants in the two countries.

As for the cross-country differences in risk perceptions, Table 3 shows that U.S. participants perceived higher risks of COVID-19 than Chinese participants at each of the three levels. Moreover, Figure 2 also reveals that the gap between participants in the two countries gradually narrowed when the reference level became further away from the personal level.

### 3.3. The Influence of Communication on Self–Other Risk Perceptions

To examine cross-country differences in the effects of communication variables on risk perceptions, we conducted moderated regression analyses. Age and gender were controlled at Step 1. Nationality and three communication variables including interpersonal communication, traditional media exposure, and new media exposure were entered at Step 2. All interactions of nationality and communication variables were entered at Step 3. Simple slope analyses were conducted to further explore the interaction effects.

**Figure 3:**
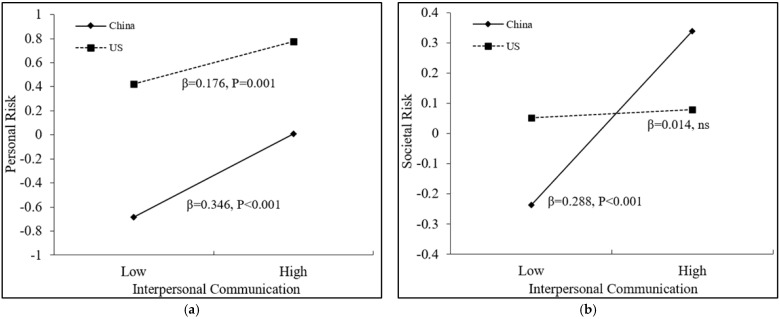

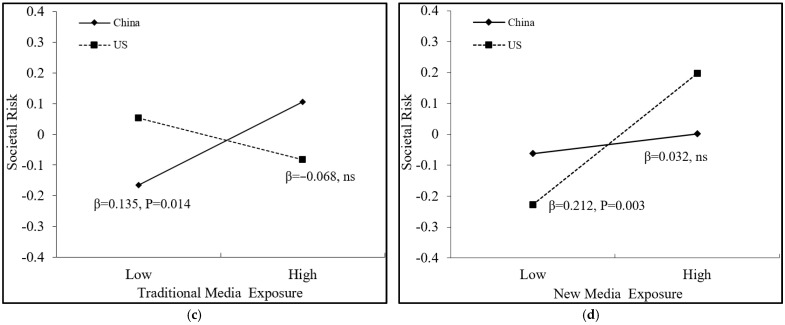
The moderating role of nationality in the association between different communication channels and self–other risk perceptions. (**a**) Nationality moderated the relation between interpersonal communication and personal risk perception; (**b**) Nationality moderated the relation between interpersonal communication and societal risk perception; (**c**) Nationality moderated the relation between traditional media exposure and societal risk perception; (**d**) Nationality moderated the relation between new media exposure and societal risk perception. Note. ns = non-significance.

As shown in Table 4, after controlling for age and gender, interpersonal communication was positively associated with perceptions of personal risk (*β* = 0.346, *p* = 0.001), group risk (*β* = 0.351, *p* = 0.001), and societal risk (*β* = 0.288, *p* = 0.001). The interaction of interpersonal communication and nationality significantly predicted perceptions of personal risk (*β* = −0.170, *p* = 0.024) and societal risk (*β* = −0.274, *p* = 0.002), which indicated the cross-country differences in the effects of interpersonal communication on personal and societal risk perceptions. As shown in Figure 3a, among Chinese college students, interpersonal communication significantly increased personal risk perception (*β* = 0.346, *p* < 0.001), whereas among U.S. college students, this correlation was attenuated (*β* = 0.176, *p* = 0.001). As shown in Figure 3b, interpersonal communication significantly increased societal risk perception among participants in China (*β* = 0.288, *p* < 0.001), whereas this correlation was non-significant among participants in the United States (*β* = 0.014, *p* = 0.825). In addition, interpersonal communication significantly increased group risk perception in China (*β* = 0.351, *p* < 0.001) and the United States (*β* = 0.220, *p* = 0.001), and the magnitude of effect tended to be higher in China, although it was not statistically significant. These results suggest that interpersonal communication may be more effective in increasing risk perceptions of Chinese college students.

As shown in Table 4, traditional and new media exposure had no significant main effect on perceptions of personal risk (*β* = 0.018, *p* = 0.695; *β* = −0.029, *p* = 0.512) or group risk (*β* = −0.069, *p* = 0.201; *β* = 0.092, *p* = 0.071). Traditional media exposure was positively associated with risk perception only at the societal level (*β* = 0.135, *p* = 0.024). The two interaction terms of traditional and new media exposure with nationality significantly predicted societal risk perception (*β* = −0.203, *p* = 0.010; *β* = 0.180, *p* = 0.046), which indicated the cross-country differences in the effects of traditional and new media exposure on societal risk perception. As shown in Figure 3c, traditional media exposure significantly increased societal risk perception for Chinese college students (*β* = 0.135, *p* = 0.014) but not for U.S. college students (*β* = −0.068, *p* = 0.283). As shown in Figure 3d, new media exposure significantly increased societal risk perception for U.S. college students (*β* = 0.212, *p* = 0.003) but not for Chinese college students (*β* = 0.032, *p* = 0.559). These results suggested that traditional media exposure was more effective in increasing the societal risk perception of Chinese college students and new media exposure was more effective in increasing the societal risk perception of U.S. college students.

## 4. Discussion

The present study examined the characteristics, influential factors, and cultural differences of self–other risk perceptions of COVID-19 at three levels (i.e., personal, group, and societal level) among college students in China and the United States. The results showed that (a) participants’ perceived risk of COVID-19 to self and to collective others showed an “ascending pattern” in the two countries, which suggested that the risk perception ascended when the reference level was further removed from the self; (b) U.S. college students perceived higher risks of COVID-19 than Chinese college students at each of the three levels; (c) interpersonal communication was positively associated with risk perceptions at three levels, and the magnitude of the effect was higher among Chinese participants than among U.S. participants; (d) traditional media exposure increased societal risk perception only for Chinese college students; (e) new media exposure increased societal risk perception only for U.S. college students.

### 4.1. Self–Other Risk Perceptions of COVID-19

In line with the opinion of “optimistic bias” [16,17], our results showed that college students perceived higher levels of risk to others than to themselves in both China and the United States, supporting H1. Specifically, the perception of societal risk was higher than that of group risk, and the perception of group risk was higher than that of personal risk, reflecting the “ascending pattern” across the three levels. The characteristics of self–other risk perceptions of COVID-19 were similar to perceptions of other health-related events, such as H1N1 [9] and skin cancer [10]. Our findings expand the current literature about risk perceptions into the context of the COVID-19 pandemic, a global risk event, especially from a comparative perspective.

As for the cross-country differences in self–other risk perceptions, our results showed that Chinese college students perceived lower risks of COVID-19 than U.S. college students at three levels, which supported H2. This finding is consistent with a previous study that investigated risk perceptions of COVID-19 in 112 countries and found that the risk perception of Chinese people was almost the lowest among these countries [53]. In addition, the results suggested that the gap between participants in the two countries became narrower when the social distance was further removed from the self. 

### 4.2. The Influence of Communication on Self–Other Risk Perceptions

Combining the results of regression and simple slope analyses, this study showed that interpersonal communication positively predicted risk perceptions at three levels, which confirmed H3a. Media exposure contributed only to the increased risk perception of the whole society, which confirmed H3b. The results support the impersonal impact hypothesis [32], showing that mass media have impersonal effects on individuals’ perceived risk to the collective others rather than to themselves. However, Tyler and Cook [32] also implied that media may affect the perceived risk to oneself under some conditions. Later, Snyder and Rouse [52] suggested that, if the media content is highly personally relevant to oneself, media exposure may exert a positive effect on personal risk perception. However, this assumption has rarely been supported by empirical research. Morton and Duck [10] found that the impact of newspaper exposure on personal risk perception was significant only for those who reported greater dependence on newspapers for health-related information.

As for the cross-country differences in the influence of communication factors on self–other risk perceptions, the simple slope analyses showed that interpersonal communication was more effective among college students in China. Specifically, the magnitude of its effect was significantly higher in China for personal and societal risk perceptions. It tended to be higher for group risk perception, although it did not reach statistical significance. The results confirmed H4a, revealing that Chinese college students relied more on social interactions to gain information. Social interactions gained more attention and trust, and thus were more influential in increasing the risk perceptions of Chinese participants. Such interactions with high personal relevance not only increased the perceived risk to themselves but could also be shared with the collective others.

Traditional and new media exposure had different effects on societal risk perception among participants in the two countries. Specifically, traditional media were effective only in China and new media were effective only in the United States, which partially supported H4b and H4c. The absence of the traditional media effect in the United States and the new media effect in China was not consistent with our hypotheses and the findings of previous studies. For example, Han et al. [9] found that SNS exposure was a significant predictor for the societal risk perception of H1N1 in China. Our findings may show the dissimilarity and specificity in the context of COVID-19, which is a global public health crisis with many rumors and misrepresentations of information spreading in multiple media channels. The mainstream values and ways to deal with the pandemic are substantially different in multiple media platforms in different countries. Such special characteristics of the COVID-19 pandemic as well as the cross-country differences in the most prominent media channels may lead to the differential effect of multiple media channels in the two countries.

### 4.3. Limitations and Future Studies

There are some limitations to this study. First, this study drew on a sample of Chinese and U.S. college students aged 17 to 30 years, representing the young and well-educated segment of the whole population. Thus, the generalizability of the findings may be limited. The structure and formation mechanism of risk perceptions may be different for people of different ages [54,55]. Future studies could investigate groups that represent other developmental stages to obtain a comprehensive understanding of the general population. Second, there are fewer participants recruited from the United States than those from China and the unbalanced sample sizes may pose a bias on the results. Future studies would try to recruit approximately equal numbers of participants from the two countries. Third, the results of this cross-sectional study are insufficient to suggest causality. Although Tyler and Cook [32] conducted experimental studies and revealed the impact of mass media on risk perception, the reverse predictive path cannot be excluded, which indicates the potential positive impact of risk perceptions on individuals’ information-searching behaviors. In other words, there may be reciprocal and dynamic relationships between risk perceptions and communication factors. Future studies could use longitudinal designs to test this hypothesis. Fourth, in this study, communication factors were quantified as the amount or frequency of interpersonal communication and media exposure, regardless of the specific content [56]. Future studies could explore the differential effects of media content on self–other risk perceptions. Finally, previous studies have shown that communication channels will amplify individuals’ risk perceptions more saliently when people actively seek rather than passively receive risk-related information. In other words, motivation and interest may influence the effect of media exposure [57]. Future studies could explore some potential moderators of the relationship between media exposure and risk perceptions of COVID-19, such as media dependence [10], attention in media [9], and trust in media [58].

### 4.4. Theoretical and Practical Implications

This study provides theoretical and practical implications to foster individuals’ risk perceptions. On the one hand, the present study gives clear conceptual and operational definitions of perceived risk to self and to others. Specifically, this study separately measured individuals’ perceived risk to themselves (personal level), their peers (group level), and average people in their own countries (societal level), showing that the “optimism bias” and the “ascending pattern” also apply to the context of the COVID-19 pandemic, a global risk event. Furthermore, to our knowledge, this study is among the first of its kind to explore the cross-country differences in the relationship between communication and self–other risk perceptions of COVID-19. Based on a comparative perspective, the results suggest that multiple communication channels have distinct effectiveness and importance in shaping risk perceptions in different countries. Our findings help to describe a comprehensive picture of the characteristics and formation mechanisms of risk perceptions from a global perspective.

Our findings also provide scientific guidance for policymakers in different countries to implement effective measures to shape individuals’ risk perceptions in relation to preventive behaviors. The results suggest that interpersonal communication is a stable and effective factor to increase individuals’ risk perception at three levels, both in China and the United States. A previous study showed that compared with mass media channels, interpersonal communication has a stronger association with individuals’ risk perceptions and preventive behaviors [36]. Thus, health campaigns should encourage the public to proactively discuss information related to the COVID-19 pandemic with their family members and friends.

Moreover, the media play leading roles in the fight against the COVID-19 pandemic. Traditional and new media exposure differentially affected societal risk perception in the two countries, with the former being more influential in China and the latter being more influential in the United States, suggesting that different countries pay more attention to different media channels. However, this does not exclude the new media effect in China or the traditional media effect in the United States. Under some conditions, both types of media may exert effects. Governments should work to improve public trust in media by challenging multiple media channels to disseminate scientific and authoritative information. Our findings can help policymakers and media practitioners in different countries to adjust strategies through various communication platforms to engage the public to combat the COVID-19 pandemic more effectively.

## 5. Conclusions

This comparative study focused on the differential impact of multiple communication channels on self–other risk perceptions of COVID-19 among college students in China and the United States. We distinguished perceived risk to self from perceived risk to others and subdivided risk perceptions into three levels: personal, group, and societal. The results revealed an “ascending pattern” of risk perceptions along the three levels in the two countries. Interpersonal communication was positively correlated with risk perceptions at three levels in the two countries, and the magnitude of the effect was higher among Chinese college students than among U.S. college students. Traditional media exposure was positively correlated with societal risk perception only for Chinese college students. New media exposure was positively correlated with societal risk perception only for U.S. college students. It appears that interpersonal communication and traditional media exposure are more effective with Chinese college students, whereas new media exposure is more effective with U.S. college students. Our findings describe a comprehensive picture of the characteristics, formation mechanisms, and cultural differences of self–other risk perceptions of COVID-19, and also provide practical guidance for policymakers and media practitioners in different countries to implement effective measures through various communication platforms to engage the public to combat the COVID-19 pandemic.

## Figures and Tables

**Table 1:** Means, standard deviations, and correlations between variables.

Variables	*M*	*SD*	1	2	3	4	5	6	7	8
1. Age	20.83	2.89	—							
2. Gender	—	—	0.30 ***	—						
3. Nationality	—	—	0.59 ***	0.29 ***	—					
4. Interpersonal communication	2.90	0.76	0.32 ***	0.22 ***	0.40 ***	—				
5. Traditional media exposure	3.39	0.91	−0.10 **	0.08 *	−0.21 ***	0.22 ***	—			
6. New media exposure	3.98	0.79	0.14 ***	0.18 ***	0.20 ***	0.45 ***	0.45 ***	—		
7. Personal risk perception	2.74	0.86	0.37 ***	0.25 ***	0.58 ***	0.47 ***	−0.03	0.22 ***	—	
8. Group risk perception	2.92	0.84	0.30 ***	0.16 ***	0.44 ***	0.42 ***	−0.05	0.23 ***	0.72 ***	—
9. Societal risk perception	3.53	0.76	0.10 **	0.20 ***	0.11 **	0.27 ***	0.16 ***	0.23 ***	0.42 ***	0.51 ***

Note: Gender (0 = male, 1 = female). Nationality (0 = China, 1 = USA). * *p* < 0.05, ** *p* < 0.01, *** *p* < 0.001.

**Table 2:** Repeated measures ANOVA and within-subjects contrasts tests on risk perceptions at three levels in two countries.

Levels Contrast	China (*n* = 498)					United States (*n* = 292)				
	*M (SD)*	*df*	*F*	*p*	Partial Eta Squared	*M (SD)*	*df*	*F*	*p*	Partial Eta Squared
Personal vs. Group	2.36 (0.71) 2.63 (0.80)	1497	93.32	<0.001	0.16	3.38 (0.70) 3.40 (0.68)	1291	0.24	=0.624	0.01
Group vs. Societal	2.63 (0.80) 3.47 (0.80)	1497	580.85	<0.001	0.54	3.40 (0.68) 3.66 (0.66)	1291	37.21	<0.001	0.11

**Table 3:** Independent *t*-tests on risk perceptions at three levels between China and the United States.

Levels	China (*n* = 498)	United States (*n* = 292)	*t* Value	*df*	*p* Value
	*M (SD)*	*M (SD)*			
Personal	2.36 (0.71)	3.38 (0.70)	−19.71	788	<0.001
Group	2.63 (0.80)	3.40 (0.68)	−14.45	788	<0.001
Societal	3.47 (0.80)	3.66 (0.66)	−3.37	788	=0.001

**Table 4:** Linear regression models on predictors of risk perceptions at three levels.

Step	Predictors	Personal Risk		Group Risk		Societal Risk	
		*β*	*SE*	*β*	*SE*	*β*	*SE*
1.	Age	−0.004	0.013	−0.002	0.013	−0.005	0.013
	Gender	0.128 *	0.064	0.007	0.070	0.280 **	0.080
2.	Nationality	0.939 **	0.089	0.621 **	0.086	0.015	0.091
	Interpersonal communication	0.346 **	0.047	0.351 **	0.055	0.288 **	0.057
	Traditional media exposure	0.018	0.047	−0.069	0.053	0.135 *	0.058
	New media exposure	−0.029	0.045	0.092	0.049	0.032	0.057
3.	Interpersonal communication × nationality	−0.170 *	0.073	−0.131	0.079	−0.274 **	0.087
	Traditional media exposure × nationality	−0.079	0.070	−0.066	0.073	−0.203 *	0.078
	New media exposure × nationality	0.085	0.076	−0.063	0.079	0.180 *	0.090
	Adj. *R^2^*	0.400 *		0.275 *		0.118 ***	

Note: Gender (0 = male, 1 = female). Nationality (0 = China, 1 = USA). Beta weights, standardized error, and adjusted *R^2^* are from the final regression equation with all predictors in the model. *n* = 790. * *p* < 0.05, ** *p* < 0.01, *** *p* < 0.001.

## Data Availability

All data generated or analyzed during the present study are available from the corresponding author on reasonable request.
